# Comparison of the Controlled Atmosphere Treatment for Submerged and Solid-State Fermentation of *Inonotus obliquus*

**DOI:** 10.3390/foods13142275

**Published:** 2024-07-19

**Authors:** Hsin-Jung Chen, Yuh-Shuen Chen, Kuo-Min Lin, Shuo-Wen Tsai, Mei-Jine Liao, Chia-Sheng Yeh, Shih-Lun Liu

**Affiliations:** 1 Department of Food Science & Technology, Central Taiwan University of Science and Technology, Beitun District, Taichung 406, Taiwan; tpsf103@gmail.com; 2 Department of Food Science and Technology, Hungkuang University, Shalu District, Taichung 433, Taiwan; chenys@sunrise.hk.edu.tw; 3 Department of Food Science and Technology, Chia Nan University of Pharmacy & Science, Rende District, Tainan 717, Taiwan; kmlin1980@mail.cnu.edu.tw; 4 Department of Food Science and Biotechnology, National Chung Hsing University, South District, Taichung 402, Taiwan; tsaishuowen@nchu.edu.tw (S.-W.T.);; 5 Department of Hospitality Management, Southern Taiwan University of Science and Technology, Yungkang District, Tainan 710, Taiwan; 6 Department of Nutrition, China Medical University, Beitun District, Taichung 404, Taiwan; 7 Department of Food Nutrition and Health Biotechnology, Asia University, Wufeng District, Taichung 413, Taiwan

**Keywords:** *Inonotus obliquus*, submerged fermentation, solid-state fermentation, controlled atmosphere treatment, PCA

## Abstract

In this study, a controlled atmosphere (CA) treatment was used in the submerged (SM) and solid-state (SS) fermentation of *Inonotus obliquus* to determine the optimal conditions. The goal was to accelerate the artificial fermentation to obtain *I. obliquus* as an ingredient for dietary supplements. The results indicated that when CA treatment was used, the SM and SS fermentation of *I. obliquus* yielded polysaccharide and betulinic acid contents 2–2.5 times higher than those obtained when such treatment was not used. The two fermentation methods yielded similar outcomes in terms of DPPH scavenging ability, bioactivity, and antioxidant activity. Although SS fermentation yielded highly bioactive fruiting bodies when the period of fermentation was extended to 60 days, the mycelia produced by SM reached a similar bioactivity quality with only 30 days of fermentation. It was indicated that SM fermentation is more economically feasible than SS fermentation in the production of *I. obliquus*.

## 1. Introduction

*Nations obliquus* (Bai-Hua-Rong, Hua-Shu-Gu, Hua-Yan-Sian-Kong-Chun, and Shie-Kuan-Sian-Kong-Chun in Chinese [1]; Chaga in Russian; Kabanoanatake or Chaga in Japanese; *Fuscoporia obliqua* (Pers.:Fr.) [2]) is a white-rot fungus belonging to the *Hymenochaetaceae* [3]. *I. obliquus* is often found as a sterile conk (sclerotium) [4], is restricted to cold habitats, and grows slowly [5]. The size of *I. obliquus* ranges from 5 to 40 cm in diameter, and the fissured surface is irregularly cracked [6].

The chemical constituents isolated from Birch Mushroom include polysaccharides [7], triterpenoids [8,9], steroids [10], aromatic compounds [11], etc., along with folic acid derivatives, small amounts of glycoproteins, amino acids, steroids, alkaloids, melanin, and lignin compounds [12]. *I. obliquus* has long been used in Eastern European countries such as Russia for the treatment of various cancers [13], and it has also been used to treat tuberculosis, gastrointestinal diseases, cardiovascular diseases, ascariasis, and viral diseases [14]. Through the research of scholars, *I. obliquus* has been proved to have antioxidant [15,16], antitumor [17], hypoglycemic [18], immunomodulatory [19], antiviral [20], and other effects.

Medicinal fungi typically require several months, or even years, of solid-state fermentation to produce fruiting bodies. Submerged fermentation is suitable for the growth of mycelia because it can continuously supply nutrients and allow for the precise control of the growth environment, leading to the rapid production of numerous mycelia within weeks. Submerged fermentation can be highly efficient for the cultivation of medicinal fungi. For instance, spores of *Beauveria bassiana* can be cultivated within 2 days [21], while the mycelia of *Cordyceps militaris* can be produced in 4 days [22]. Extending the submerged fermentation period allows for the cultivation of higher quantities of *Phellinus igniarius* mycelia within 22 days [23]. Additionally, after 30 days of cultivation, it is possible to produce fruiting body-like structures of *Antrodia cinnamomea* [24]; it is possible to produce *Antrodia cinnamomea* fruiting bodies similar to those obtained through cut-log cultivation. Some researchers refer to the fruiting bodies of *Antrodia cinnamomea* cultured in Petri dishes as "pseudo-fruiting bodies" because the nutrients in the Petri dish are insufficient to support further growth of the fruiting bodies. Nevertheless, submerged fermentation is faster than solid-state fermentation and can quickly yield bioactive mycelia. However, t cannot induce the transformation of mycelia into fruiting bodies, and the bioactivity of mycelia is lower than that of fruiting bodies [25]. In terms of the bioactivity of fermentation products, solid-state fermentation is superior to submerged fermentation. Currently, when using medicinal fungi as raw materials for health foods, extraction and concentration methods are employed to enhance the content of the bioactive compounds. These processes significantly alter the concentration of the bioactive compounds in medicinal fungi. For instance, Liang et al. found that the bioactive compound content in extracts from mycelia was higher than that in extracts from fruiting bodies when different solvents were used [26]. Overall, both solid-state and submerged fermentation have their respective advantages and disadvantages [27]. Health food manufacturers can choose the appropriate fermentation method for medicinal fungi based on their specific needs.

This study introduced an innovative controlled atmosphere cultivation model aimed at enhancing the effectiveness of both solid-state and submerged fermentation. Both the growth and functional ingredient content of *I. obliquus* undergoing solid-state and submerged fermentation were compared to determine the optimal fermentation method that can produce functional mycelia or fruiting bodies of *I. obliquus*. The ultimate goal was to obtain high-quality *I. obliquus* as a raw material for various types of dietary supplements.

## 2. Materials and Methods

### 2.1. Experimental Design

The controlled atmosphere treatment refers to mixing N_2_ and O_2_ at a 50:50 ratio and then introducing this mixture into solid-state and submerged fermentation equipment through a small tube at a constant flow rate of 10 vvm (m^3^/(m^3^·min)). This process ensures that the gas composition within the equipment maintains the 50:50 ratio of N_2_ and O_2_. Samples were taken on the 10th, 20th, and 30th days for the determination of biological activity and antioxidant power during the solid-state and submerged fermentation modes, while the solid culture continued to be cultured until the 60th day, and samples were also taken every 10 days for the above analysis.

### 2.2. Fungal Material

The strain of *I. obliquus* was collected in China. The species name was identified by the Bioresource Collection and Research Center, Taiwan. The strain was kindly provided by the functional foods laboratory, department of food science and technique, HungKuang University, Taichung, Taiwan, ROC.

### 2.3. Strain Activation and Purification

The inoculation loop was used to dip the strain and the streaking method was used to culture the fungi on the inoculation loop on a Potato Dextrose Agar (PDA) plate culture medium, with three zones of purification cultivation. After incubation at 18 °C for 3~10 days, the mycelium will appear on the plate, and the purification will be completed three times in a row, completing the purification and activation of *I. obliquus*.

### 2.4. Scale-Up of Strain

The purified mycelium was taken from the plate culture medium. Then, 3 loops of mycelium were inoculated into a liquid culture medium (D(+)–glucose 0.25 g, peptone 4 g, sodium chloride 2.5 g, yeast extract 0.75 g, Reverse Osmosis (RO) water 250 mL), placed in a constant-temperature rotary shaking incubator, and incubated at 18 °C, 125 rpm, for 7 days under sunlight.

### 2.5. Submerged Fermentation of I. obliquus

In the submerged culture section, the cultured strain was inoculated into a 10 L fermentor (14 L, MS F2000, Major Science, Taoyuan, Taiwan) with 2% of the inoculum, and the nutrient composition of the culture solution was D-(+)-glucose 1 kg, peptone 160 g, sodium chloride 100 g, yeast extract 30 g, corn kernel 400 g, mulberry wood powder 600 g, RO water 10 L. The cultures were incubated at 18 °C for 30 days under light protection with the CA system (air injection into the cultivation system was conducted through the forced-outlet air stream) and the normal air system (air was injected into the cultivation system at 150 rpm agitation), and samples were taken every 10 days for analysis [28].

### 2.6. Solid-State Fermentation of I. obliquus

The contents of the spent substrate were taken after harvesting king oyster mushrooms. Then, corn grain (10%) and mulberry wood powder (15%) were weighed relative to the total weight of the substrate contents, thoroughly mixed, and utilized used as the medium for solid-state culture. A total of 2 mL (2%) of the strain stock solution was taken and 100 g of the solid-state medium was inoculated onto the surface and then incubated in an incubator with the CA treatment gases injected and under normal atmosphere for 60 days, taking samples every 10 days for analysis [29].

### 2.7. Freeze-Dried Powder Preparation

After submerging the cultured mycelium, the submerged culture medium was filtered and removed, and the mycelium was freeze-dried to obtain the liquid culture freeze-dried powder. For the solid-state-cultured mycelium and fruiting bodies, after freeze-drying the fruiting bodies and mycelium, they were peeled off from the grain medium, and then the peeled fruiting bodies and mycelium were powdered to obtain the solid-state culture freeze-dried powder.

### 2.8. Polysaccharide Contents

A total of 0.1 g of the freeze-dried powder of *I. obliquus* was weighed into a 50 mL centrifuge tube, and 50 mL of deionized water was added. This was heated at 100 °C for 2 h, then cooled and centrifuged at 2500 rpm for 5 min. The upper layer of the liquid was collected and quantitatively reduced to 100 mL. A total of 5 mL of the upper layer of the liquid was added to 20 mL of 95% alcohol, shaken to mix, placed at 4 °C for 12 h, and then centrifuged at 2200 rpm for 20 mins. The upper layer of liquid was poured off, 5 mL of deionized water was added to re-dissolve, and then the polysaccharide solution of *I. obliquus* was obtained. To determine the polysaccharide content by the phenol-sulfuric acid method, 0.5 mL of the polysaccharide solution of *I. obliquus* was taken, 0.5 mL of 5% phenol solution was added, and 2.5 mL of concentrated sulfuric acid was added. The mixture was shaken and mixed well, then allowed to stand at room temperature for 15 min. The absorbance value of the 490 nm wavelength was measured by a spectrophotometer, and then the concentration was converted using a glucose standard curve. The analysis was replicated in triplicate [30].

### 2.9. Betulinic Acid Contents

Betulinic acid, an indicator component of the triterpenoids of *I. obliquus*, was chosen as a representative indicator component. An amount of 0.1 g of freeze-dried powder of *I. obliquus* was weighed, 10 mL of methanol was added, and the extract was extracted by ultrasonic extraction for 60 min. After extraction, 10 mL of methanol was used for quantitative analysis, 1 mL of the extract was centrifuged at 12,000 rpm for 3 min, and the supernatant was filtered through 0.45 μm filter membranes for analysis using HPLC (L-2130, L-2400, D-2000, Hitachi High-Technologies Corporation, Tokyo, Japan). The analysis was replicated in triplicate [31].

Column: LiChrospher 100 RP−18 (250 × 4.6 mm, 5 um, Merck, Rahway, NJ, USA).
Injection volume: 20 µL.
Temperature of column: 30 °C.
Mobile phase: Methanol: 0.2% formic acid = 85:15.
Flow rate: 1 mL/min.
Detection wavelength: 210 nm.

### 2.10. DPPH’s Radical Scavenging Potential

DPPH (α,α-diphenyl-β-picrylhydrazyl)’s radical scavenging potential was assayed as described in [32,33]. Briefly, 2.0 ml of 0.1 mM DPPH in ethanol was added to 2.0 mL of each sample. The mixture was shaken violently and incubated at 30 °C for 30 min, followed by estimating its absorbance at 517 nm [34]. Ethanol was used as the blank control. The scavenging activity was expressed as the percentage inhibition of the DPPH radicals by 1 g of the total phenolic compounds (melanin is not included), which was calculated from [1 − (A1/A0)] × 100, where A1 and A0 are absorbencies of the sample and the control, respectively. The analysis was replicated in triplicate.

### 2.11. Statistical Analysis

Values are presented as the mean ± standard deviation (*n* = 3). Analysis of variance (ANOVA) was conducted in the SAS software version 8.0 (SAS Institute, Cary, NC, USA) with a mixed model involving repeated measures as random effects and fermentation methods (submerged vs. solid-state fermentation), air injection methods (controlled atmosphere treatment vs. normal air injection), and fermentation durations in days (10 vs. 20 vs. 30 vs. 40 vs. 50 vs. 60 days) as main effects. Subsequently, a *t* test was conducted to determine whether different fermentation methods, air injection methods, and fermentation durations were associated with significantly varying outcomes, with the significance level set at *p* < 0.05. Finally, principal component analysis was conducted using a covariance matrix model in XLSTAT in Microsoft Excel version 2019 (Microsoft, Redmond, WA, USA).

## 3. Results

### 3.1. Significance of Different Treatments

Table 1 presents the mixed model results of the effect of each factor on the bioactive ingredients of *I. obliquus*. A mixed-model ANOVA treats repeated measures as random effects, enabling a precise assessment of the effects of individual variables on the outcome [29]. As shown in Table 1, the fermentation method (submerged vs. solid-state fermentation) affected the bioactivity of *I. obliquus*, but the effect was nonsignificant. By contrast, the air injection method and fermentation duration significantly affected the bioactivity of *I. obliquus*. The interactions between the variables of the polysaccharide content and betulinic acid content were also investigated. The results indicated that the interaction effect between the fermentation method and fermentation duration on the polysaccharide content in *I. obliquus* was nonsignificant (*p* > 0.05) but the effect on the betulinic acid content was significant (*p* < 0.05). In the following section, the changes that occurred in the contents of polysaccharides and betulinic acid over the course of fermentation are discussed.

### 3.2. The Polysaccharide Contents of I. obliquus

**Figure 1:**
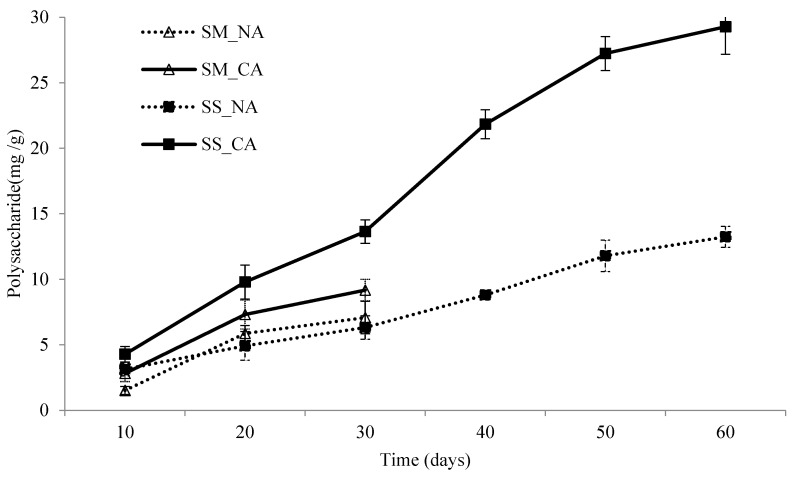
The influence on polysaccharide contents by fermentation condition. SM: submerged fermentation; SS: solid-state fermentation; NA: normal air system; CA: controlled air system. The SD bars are presented as mean ± SD, N = 3.

Figure 1 presents the content of polysaccharides, sampled at 10-day intervals, in *I. obliquus* undergoing submerged and solid-state fermentation at 18 °C. During submerged fermentation, the mycelia in the fermentor began exhibiting signs of deterioration after 30 days because of the lack of nutrients. Consequently, fermentation beyond this time point was inappropriate. During solid-state fermentation, the fruiting bodies kept growing after 30 days. Therefore, the fermentation process was extended to 60 days, until signs of deterioration were observed because of the lack of nutrients. During the extended fermentation period, the sampling frequency remained at once every 10 days.

As shown in Figure 1, the content of polysaccharides in *I. obliquus* undergoing solid-state and submerged fermentation substantially increased with increasing fermentation time. The controlled atmosphere treatment resulted in a 1.3- and 2.2-fold increase in the content of polysaccharides in *I. obliquus* undergoing solid-state and submerged fermentation, respectively. These results confirmed the effectiveness of the controlled atmosphere treatment in increasing the content of polysaccharides during fermentation.

Both submerged fermentation and solid-state fermentation are commonly used to obtain fungal products. During their growth, fungi secrete polysaccharides of various features [35]. When *I. obliquus* was exposed to natural hydroxyl groups, they produced oxidizing substances, including polysaccharides and phenols. According to Li et al., fungi have a strong 2,2-diphenyl−1-picrylhydrazyl (DPPH) inhibition capability, which is linked to the hydrogen-donating property of the hydroxyl groups in polysaccharides [36]. In *I. obliquus*, polysaccharides are primarily composed of heteroglycans and homoglucans and are therefore more effective against tumors than those produced by the mycelia or fruiting bodies of other fungi [37]. In this study, during the submerged and solid-state fermentation of *I. obliquus*, the controlled atmosphere treatment was conducted using inflow air containing 50% oxygen, which is considerably higher than the concentration of oxygen in normal air. Given the aerobic nature of *I. obliquus*, this high concentration of oxygen in the controlled atmosphere presumably increased the content of polysaccharides, as verified by an increase in polysaccharide content to 9.17 and 13.64 mg/g in *I. obliquus* undergoing submerged and solid-state fermentation, respectively, on day 30. Notably, the polysaccharide content achieved with solid-state fermentation was 1.48 times higher than the polysaccharide content achieved with submerged fermentation, and both contents were significantly higher than that obtained by Xu et al. (1.1 mg/g) [38]. Calculations based on dry mycelial weight revealed that the content of polysaccharides achieved with submerged fermentation reached 537.9 mg/g, which is significantly higher than the contents obtained by Zheng et al. (45 mg/g) and Hu et al. (240 mg/g) [15,30].

On day 60 of solid-state fermentation, the content of polysaccharides reached 2.1 times that achieved on day 30, presumably because of the close bond between mycelia and certain genes during the long fermentation period, as indicated by the structures of the fruiting bodies and mycelia. Although the cultured fruiting bodies were automatically detached from the culture medium during the freeze-drying and grinding process, the mycelia did not undergo the same process. After 30 days, starch was observed in the solid-state culture medium, presumably because of the high content of polysaccharides. In addition, the use of the phenol–sulfuric acid method to quantify the content of polysaccharides may have been misleading because the starch present in the medium, despite not containing polysaccharides, was decomposed into monosaccharides.

According to the interaction results of the mixed-model analysis in Table 1, both the controlled atmosphere treatment and fermentation duration, despite having major effects, exhibited nonsignificant interaction effects on the content of polysaccharides throughout the fermentation process. The interaction effect between the fermentation method and the controlled atmosphere treatment on the content of polysaccharides (*p* = 0.04) was also significant. By contrast, the interaction effect between the air injection method, fermentation duration, and fermentation method was nonsignificant (*p* = 0.05), indicating that the effects of these three variables on the content of polysaccharides were independent of each other.

### 3.3. The Betulinic Acid Contents of I. obliquus

**Figure 2:**
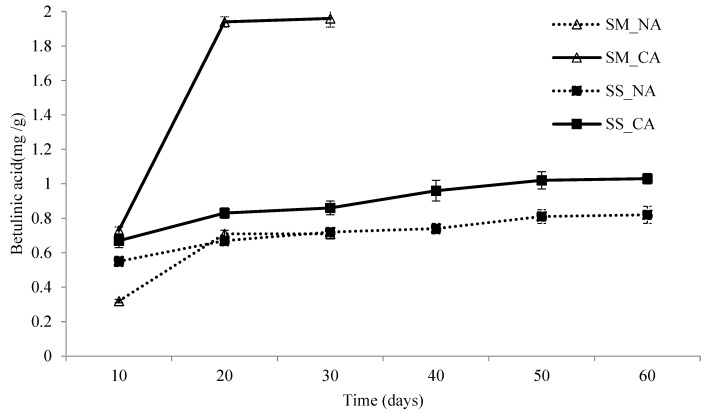
The influence on betulinic acid contents by fermentation condition. SM: submerged fermentation; SS: solid-state fermentation; NA: normal air system; CA: controlled air system. The SD bars are presented as mean ± SD, N = 3.

Figure 2 presents the contents of betulinic acid, sampled at 10-day intervals, in *I. obliquus* undergoing submerged and solid-state fermentation at 18 °C. In both fermentation methods, the content of betulinic acid substantially increased over time, with this increase slowing down after 20 days of fermentation. With the controlled atmosphere treatment, the content of betulinic acid in *I. obliquus* undergoing submerged fermentation and solid-state fermentation was 2.8 and 1.2 times, respectively, higher than when such treatment was not used. These results confirmed the effectiveness of the controlled atmosphere treatment in increasing the content of betulinic acid during fermentation. During the solid-state fermentation process, the amount of betulinic acid obtained in the controlled atmosphere was similar to that obtained in normal air.

When the duration of solid-state fermentation was extended by 30 days, the content of betulinic acid increased 1.2 times. Consequently, the production of triterpenoids (e.g., betulinic acid) in *I. obliquus* was rapid only during the first 20 days of fermentation and slowed down thereafter.

Betulinic acid is a triterpenoid that serves as a key functional ingredient in *I. obliquus* [39]. Betulin is a precursor of betulinic acid that functions as an essential active ingredient. Both betulinic acid and betulin exhibit various types of bioactivities, including antitumor activity, anti-human immunodeficiency virus activity, anti-inflammatory activity, and anti-microorganism activity. Among these types of bioactivities, the antitumor activity of betulinic acid and betulin attracts the most attention, globally, because both substances have shown favorable outcomes in the treatment of various types of cancer [40]. Fermentation typically involves the metabolism of betulin into betulinic acid, which outperforms betulin in terms of functionality and utility [41]. Therefore, in this study, betulinic acid was comprehensively analyzed. When the controlled atmosphere treatment was used, submerged and solid-state fermentation of *I. obliquus* yielded 1.96 and 0.86 mg/g betulinic acid, respectively, after 30 days. The content of betulinic acid obtained with submerged fermentation was converted, based on mycelium weight, into 188.7 mg/g, which is higher than the content obtained by Bai et al. from mycelia cultivated using a fermentor [41].

According to the interaction results of mixed-model analysis (Table 1), the interaction effect of the controlled atmosphere treatment and fermentation duration on the content of betulinic acid was the most pronounced. In addition, the fermentation method had a nonsignificant effect on the content of betulinic acid. Moreover, the interaction effect between the air injection method, fermentation duration, and fermentation method was nonsignificant, showing that these three variables had independent effects on the content of betulinic acid.

### 3.4. The DPPH Radical Scavenging Potential of I. obliquus

**Figure 3:**
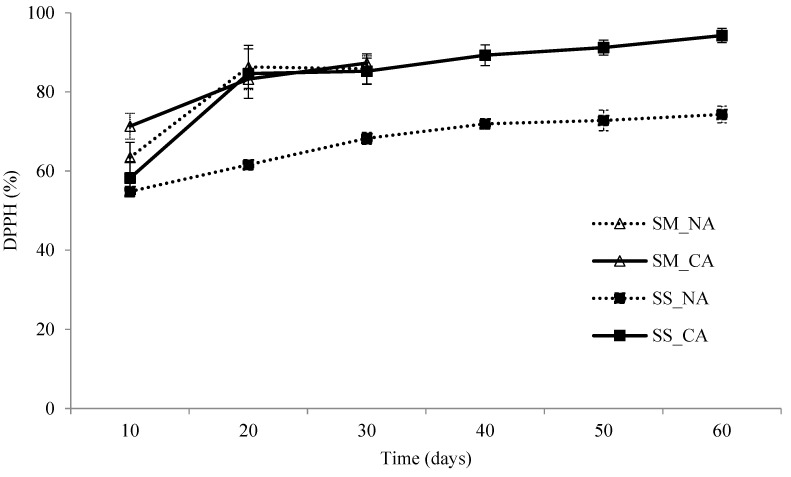
The influence on DPPH radical scavenging potential by fermentation condition. SM: submerged fermentation; SS: solid-state fermentation; NA: normal air system; CA: controlled air system. The SD bars are presented as mean ± SD, N = 3.

In fungi, polyphenols exhibit a high antioxidant activity. Both betulinic acid and sterols, such as lanosterol, inotodiol, trametenolic acid, and ergosterol peroxide, have a higher antioxidant activity than that of polysaccharides and polyphenols [42]. Figure 3 presents the results of a DPPH free-radical scavenging assay conducted at 10-day intervals on *I. obliquus* undergoing submerged and solid-state fermentation at 18 °C. The results indicated that the DPPH free-radical scavenging ability of *I. obliquus* undergoing submerged and solid-state fermentation substantially increased over time, but this increase slowed down on day 20. Even when the solid-state fermentation process was extended to 60 days, the DPPH free-radical scavenging ability of *I. obliquus* was only 1.1 times higher than that achieved on day 20. This pattern is consistent with the previously discussed increase in betulinic acid content and aligns with the argument of Cui [42], who asserts that triterpenoids are the main components affecting the antioxidant activity of fungi. When the controlled atmosphere treatment was used, submerged and solid-state fermentation resulted in an increase of 2.4% and 19.3%, respectively, in the DPPH free-radical scavenging ability of *I. obliquus*. During the submerged fermentation process, the DPPH free-radical scavenging ability achieved in the controlled atmosphere was similar to that achieved when normal air was injected.

When the controlled atmosphere treatment was used, the DPPH free-radical scavenging abilities achieved after 30 days of submerged and solid-state fermentation were close to each other, at 87.32% and 84.59%, respectively. These values are higher than the percentage (60%) obtained by Xiang et al. [43] and the percentages (65% and 75%) obtained by Xu et al. [44], who cultured *I. obliquus* in flasks, and they are close to the percentage obtained by Zheng et al. (83%), who also used a fermentor to culture *I. obliquus* [5]. Therefore, injecting air from the top of a fermentor and controlling the atmosphere during solid-state fermentation may enhance the outcomes of the submerged and solid-state fermentation of *I. obliquus*.

According to the mixed-model results of the interaction effects on DPPH free-radical scavenging ability (Table 1), the interaction effect of the controlled atmosphere and fermentation duration on the free-radical scavenging ability of DPPH was nonsignificant, despite both having major effects. In addition, the fermentation method had a nonsignificant effect on the free-radical scavenging ability of DPPH. Moreover, the interaction effect between the air injection method, fermentation duration, and fermentation method was nonsignificant, showing that these three variables had independent effects on the free-radical scavenging ability of DPPH.

As shown in Figure 1, Figure 2 and Figure 3, the content of polysaccharides in *I. obliquus* doubled after 60 days of solid-state fermentation. However, the content of betulinic acid and the free-radical scavenging ability of DPPH exhibited only a minor increase after 20 days of both solid-state and submerged fermentation.

**Figure 4:**
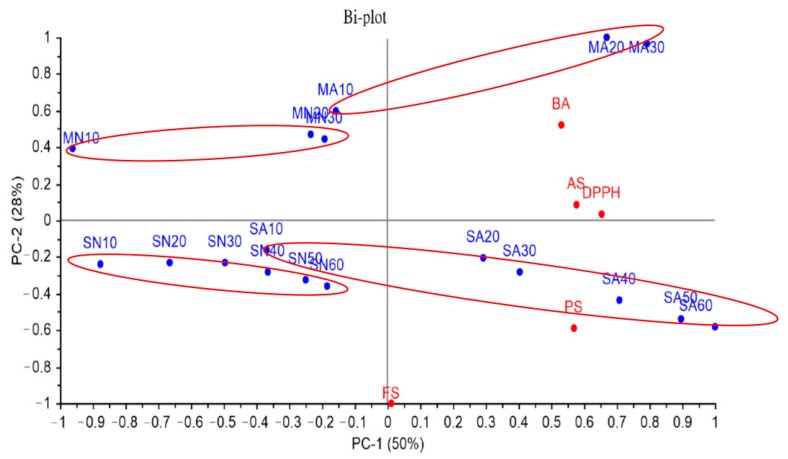
The PCA plot of different controlled atmospheres with submerged and solid-state fermentation. PS: polysaccharide; BA: betulinic acid; AS: atmosphere system; FS: forced controlled atmosphere system; SA: solid-state fermentation with controlled atmosphere system; SN: solid-state fermentation with normal atmosphere system; MA: submerged fermentation with controlled atmosphere system; MN: submerged fermentation with normal atmosphere system. Red circles: Samples with similar characteristics will be circled in the same red circle.

To identify the increasing trends of individual functional ingredients across various fermentation treatments and methods, principal component analysis was conducted (Figure 4). The first and second principal components explained 50% and 28%, respectively, of the variance in the sample, with a combined variance of 78%, showing strong explanatory effects for the two principal components [45]. Among all variables, the controlled atmosphere treatment had the strongest effect on the overall quantity of functional ingredients and was used to divide the samples, regardless of the fermentation method, into two groups. The duration of fermentation exhibited the second-strongest effect on the overall quantity of functional ingredients, and it also strongly and positively correlated with these ingredients. The content of betulinic acid in *I. obliquus* was higher with submerged fermentation than with solid-state fermentation, whereas the content of polysaccharides was higher with solid-state fermentation than with submerged fermentation. The free-radical scavenging ability of DPPH strongly and positively correlated only with the duration of fermentation and was unaffected by the method of fermentation.

Overall, the number of fruiting bodies increased when the solid-state fermentation process of *I. obliquus* was extended to 60 days. However, during the extended fermentation period, the overall improvement observed in the functionality was limited. To evaluate the economic benefits of submerged and solid-state fermentation, the content of functional ingredients and the duration of fermentation were measured. The results indicated that the overall functionality and content of functional ingredients in *I. obliquus* undergoing solid-state fermentation were lower than those achieved with two 30-day rounds of submerged fermentation. Therefore, submerged fermentation of *I. obliquus* was more economically workable than solid-state fermentation. In summary, solid-state fermentation should be used only if the purpose is to obtain *I. obliquus* fruiting bodies.

## 4. Conclusions

In this study, submerged fermentation and solid-state fermentation were used to culture *I. obliquus*. Submerged fermentation was performed to obtain mycelia, and solid-state fermentation was performed to obtain both mycelia and fruiting bodies. After 30 days of fermentation, the two methods yielded similar levels of bioactivity and antioxidant activity. Although solid-state fermentation yielded fruiting bodies within a fermentation period of 60 days, two rounds of submerged fermentation could have been performed within this period, which could have produced a larger quantity of functional ingredients. These results indicated that submerged fermentation is more economically feasible than solid-state fermentation in the production of *I. obliquus*. Through air injection, submerged fermentation may yield functional ingredients and antioxidant activity comparable to those achieved with solid-state fermentation. Therefore, optimizing the production conditions inside submerged fermentors by, for example, incorporating a fed-batch culture or a bi-stage culture and adding elicitors or precursors would increase the content of functional ingredients in *I. obliquus* to considerably exceed that achieved with solid-state fermentation.

## Figures and Tables

**Table 1:** One-way analysis of variance for different fermentation conditions (mixed model).

Source	DF	*p*-Value		
		Polysaccharide	Betulinic Acid	DPPH
FS ^a^	1	0.04	0.05	0.03
AS	1	<0.01	0.02	<0.01
FT	2	<0.01	<0.01	<0.01
FS × AS	1	0.04	0.03	0.01
AS × FT	2	0.02	0.08	0.07
FS × FT	2	0.47	0.02	0.07
FS × AS × FT	2	0.05	0.10	<0.01

^a^: FS: fermentation substrate; AS: air system; FT: fermentation time.

## Data Availability

The raw data supporting the conclusions of this article will be made available by the authors on request.
